# Functionalized Lineage Tracing Can Enable the Development of Homogenization-Based Therapeutic Strategies in Cancer

**DOI:** 10.3389/fimmu.2022.859032

**Published:** 2022-05-06

**Authors:** Catherine Gutierrez, Caroline K. Vilas, Catherine J. Wu, Aziz M. Al’Khafaji

**Affiliations:** ^1^ Department of Medicine, Harvard Medical School, Boston, MA, United States; ^2^ Department of Medical Oncology, Dana-Farber Cancer Institute, Boston, MA, United States; ^3^ Department of Molecular Biosciences, The University of Texas at Austin, Austin, TX, United States; ^4^ Division of Chemical Biology and Medicinal Chemistry, College of Pharmacy, The University of Texas at Austin, Austin, TX, United States; ^5^ Department of Medicine, Brigham and Women’s Hospital, Boston, MA, United States; ^6^ Broad Institute of MIT and Harvard, Cambridge, MA, United States

**Keywords:** tumor heterogeneity, clonal dynamics, clonal evolution, drug resistance, homogenization, cellular plasticity, functionalized lineage tracing, DNA barcoding

## Abstract

The therapeutic landscape across many cancers has dramatically improved since the introduction of potent targeted agents and immunotherapy. Nonetheless, success of these approaches is too often challenged by the emergence of therapeutic resistance, fueled by intratumoral heterogeneity and the immense evolutionary capacity inherent to cancers. To date, therapeutic strategies have attempted to outpace the evolutionary tempo of cancer but frequently fail, resulting in lack of tumor response and/or relapse. This realization motivates the development of novel therapeutic approaches which constrain evolutionary capacity by reducing the degree of intratumoral heterogeneity prior to treatment. Systematic development of such approaches first requires the ability to comprehensively characterize heterogeneous populations over the course of a perturbation, such as cancer treatment. Within this context, recent advances in functionalized lineage tracing approaches now afford the opportunity to efficiently measure multimodal features of clones within a tumor at single cell resolution, enabling the linkage of these features to clonal fitness over the course of tumor progression and treatment. Collectively, these measurements provide insights into the dynamic and heterogeneous nature of tumors and can thus guide the design of homogenization strategies which aim to funnel heterogeneous cancer cells into known, targetable phenotypic states. We anticipate the development of homogenization therapeutic strategies to better allow for cancer eradication and improved clinical outcomes.

## Introduction

Recent advances in our understanding of the molecular pathogenesis and therapeutic responses of cancer have enabled the development of potent novel therapeutic modalities across many cancer types. These strategies include targeted therapies, which seek to eradicate cancer cells by interfering with specific molecules or key cellular processes necessary for tumor survival and growth, and immunotherapies, which are designed to modulate the immune response to improve targeting and elimination of cancer cells. While these modalities have revolutionized patient outcomes often in synergy with traditional chemotherapy in many cancers, patients nonetheless continue to exhibit pre-existing or adaptive therapeutic resistance and disease recurrence. The innumerable therapeutic resistance mechanisms identified to date have underscored profound propensity and capacity for cancer to evolve (1). Tumor evolution is driven by the dynamic interplay between cellular plasticity and environmental pressures, resulting in constantly variegating subpopulations with many possible avenues for therapeutic escape (2). Current therapies inadequately address and often contribute to such heterogeneity. Certain chemotherapeutic agents, for instance, induce various forms of DNA damage that cause increased chromosomal aberrations or mutations, thus fueling heterogeneity (3–6). Targeted therapies are often chosen for their ability to selectively target cancer cells harboring a characteristic biomarker and in certain cases have revolutionized patient care (e.g., clinical introduction of imatinib, a BCR-ABL tyrosine kinase inhibitor, has more than tripled the 5-year survival rate for patients with chronic myelogenous leukemia) (7). However, this approach can fall short due to the presence of tumor subpopulations with low protein expression or mutations altering the drug-binding site, enabling therapeutic escape (8, 9). In another example, immunotherapies such as immune checkpoint inhibitors, while curative in a proportion of patients, often produce variable immune responses against different tumor lesions within the same patient. Additionally, many patients either do not respond, experience waning efficacy due to progressive T cell exhaustion, or develop resistance after treatment (i.e., *via* changes in tumor neoantigen expression and/or immunogenicity or *via* downregulation of antigen presentation pathways) (10, 11). Therefore, it is imperative that alternative novel treatment strategies are explored to address shortcomings that remain despite such recent therapeutic advances.

While the underlying genetics of a tumor often heavily influence its phenotype, the phenotypic profiles or “cell states” of a tumor have not been found to strongly associate with specific mutational patterns (12, 13). Our understanding of the interplay between tumor genetics, epigenetics, and expression profiles and the tumor microenvironment remains rudimentary; however, the field is accumulating evidence of how dynamic gene regulatory networks and various environmental pressures play central roles in modulating the diverse phenotypic cell states that individual cancer cells can occupy. As different cell states can exhibit varying sensitivities to therapy, treating a highly diverse tumor with any given single or combination therapy is unlikely to effectively address the assortment of available transcriptional states present across millions to billions of tumor constituents. This presents another basis for therapeutic clonal escape and is a formidable clinical challenge.

The recent introduction of functionalized lineage tracing approaches, capable of capturing the multi-omic characteristics of millions of clones over a treatment course, can inform a lineage- and temporally-resolved understanding of the mechanisms cancer cells employ during acute stress. This in turn potentially enables the design and application of novel tumor homogenization approaches to therapy, which aim to reduce intratumoral heterogeneity. Specifically, tumor ‘homogenizing’ agents can be screened for their ability to rationally drive a genetically and/or phenotypically heterogeneous population towards a desired, actionable set of phenotypic programs that are vulnerable to a second, known therapeutic agent (e.g., chemotherapy, targeted agent, or immunotherapy).

Herein, we describe current conceptual models of tumor evolution and highlight the limitations of existing therapeutic approaches to cancer. Further, we detail novel approaches that aim to constrain intratumor heterogeneity and thus curtail avenues of therapeutic escape. Finally, we discuss recent technological advances that hold great promise for enabling and informing therapeutic approaches such as tumor homogenization.

## Intratumoral Heterogeneity and Therapeutic Resistance

Technological advances have enabled pan-cancer sequencing efforts, resulting in the discovery of extensive genetic, epigenetic, and transcriptomic heterogeneity across and within tumors (14–16). Numerous studies demonstrate that the presence of a high degree of intratumor heterogeneity is associated with poor prognosis (14, 17–19). With increased heterogeneity is a greater likelihood that cells within the bulk tumor will exhibit differing sensitivities to therapy (e.g., a rare clone may harbor a pre-existing resistance mechanism(s) or clones may acquire drug tolerance and/or resistance throughout treatment, permitting clonal survival and expansion) (2). As we deepen our understanding of the role heterogeneity plays in therapeutic resistance, it is increasingly clear that purposefully shaping and constraining heterogeneity is likely to be fruitful.

### Evolutionary Mechanisms of Cancer

**Figure 1:**
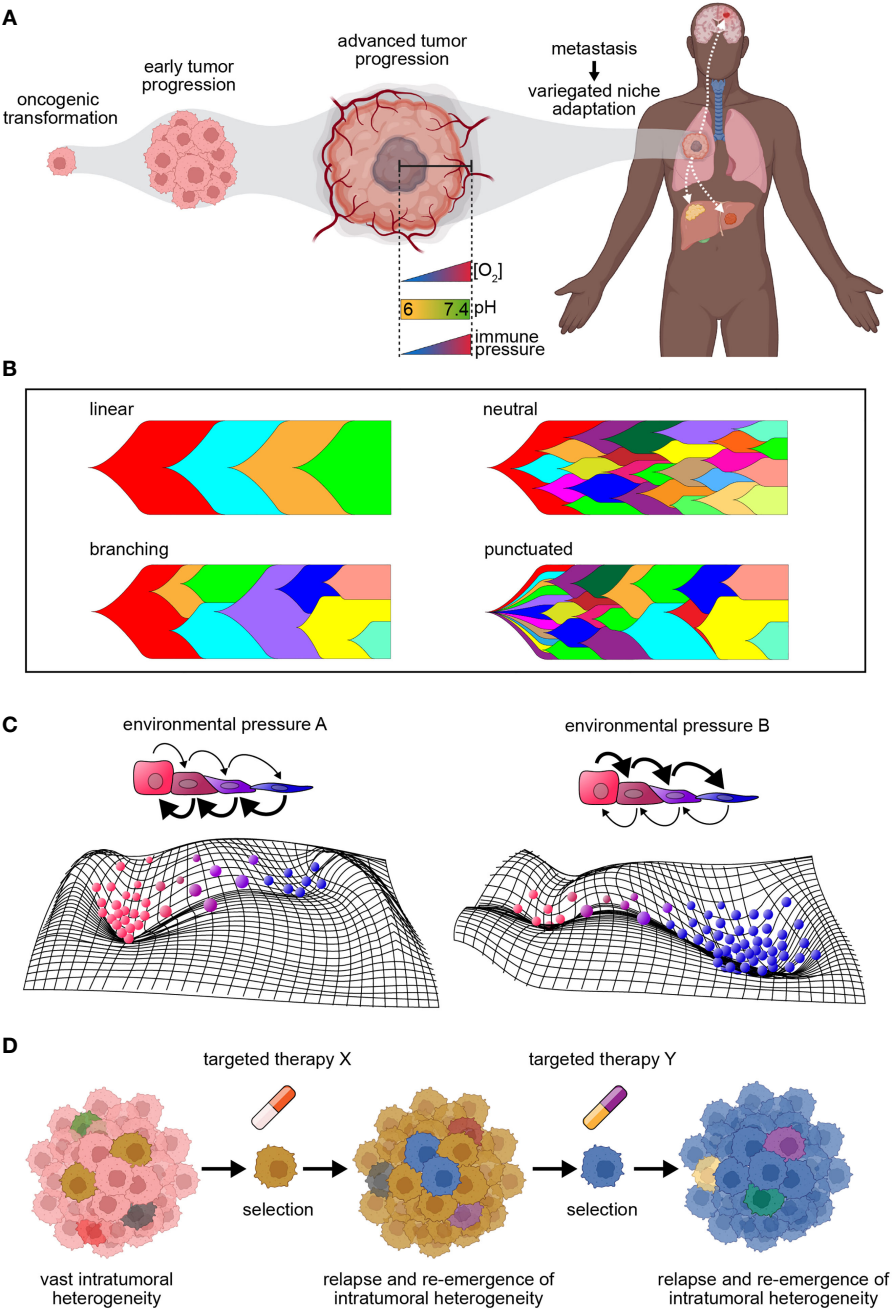
Tumor dynamics in the context of progression and therapy. **(A)** Diagram illustrating the process of tumor progression beginning from a single founding clone. Tumor progression coincides with increasing intratumoral heterogeneity, and as metastasis occurs, clones exhibit variegated niche adaptation. **(B)** Models of tumor evolution. Linear evolution = mutations acquired in a stepwise fashion with driver mutations fueling selective sweeps of clonal dominance; branching evolution = clones evolve simultaneously, selecting for increased fitness over time; neutral evolution = clonal expansion in absence of stringent selection leads to passive accumulation of genomic alterations; punctuated evolution = mutation bursts resulting in the sudden accrual of genomic changes. **(C)** Top: Schematic of cell state transitions in the presence of environmental pressures **(A, B)**. Arrows represent directionality of state transitions, where bold arrows represent increased transition rates. Color of cells corresponds to different cell states. Bottom: Representative cell state manifolds depicting influence of above environmental pressures on modulating cell state. Peaks and troughs represent cell state stability. **(D)** Model depicting key limitations of targeted therapy. Intratumor heterogeneity serves as a sustainable source of resistance, fueling the survival of clones with drug tolerance or resistance throughout treatment. This drives tumor relapse and the re-emergence of intratumoral heterogeneity, resulting in a continuous cycle.

The process of oncogenesis begins with the transformation of a single, founding neoplastic cell - a consequence of cell cycle dysregulation in conjunction with abrogated apoptotic signaling. This results in uncontrolled proliferation, tempered by resource limitation, overcrowding, and eventual toxic substrate accumulation - shaping new and local environmental conditions to be overcome (**Figure 1A**). Throughout tumor progression, individual cancer cells undergo a number of heritable molecular alterations that fuel evolution and heterogeneity. Clones with alterations that enhance cellular survival and proliferation experience increased fitness and undergo positive selection. Likewise, deleterious alterations result in decreased fitness, such that clones undergo negative selection and possible eradication from the tumor population. The resultant tumor population then is comprised of numerous subpopulations, each distinct in their abilities to access a range of advantageous cell states.

Several tumor progression models to date have been described (**Figure 1B**), including linear evolution, where mutations are acquired in a stepwise fashion and driver mutations fuel selective sweeps of clonal dominance throughout tumor evolution (20); branching evolution, where clones evolve simultaneously, resulting in multiple subclonal branches that demonstrate selection for clones with increased fitness over time within the tumor; neutral evolution, where expansion in the absence of stringent selection leads to passive accumulation of genomic alterations (e.g., the Big Bang model of colorectal tumor growth) (21); and punctuated evolution, depicted by mutation bursts or cataclysmic genomic rearrangements resulting in the sudden accrual of genomic changes (20). Irrespective of the mode of evolution in treatment-naive tumors, therapy of all types can either contribute to increased intratumoral heterogeneity or impose a selective pressure that results in the expansion of a resistant subclone (22).

While cancer has long been considered a genetic disease, where heritable DNA alterations serve as substrates for evolution (gene-centric model of evolution), it is now evident that non-genetic sources of phenotypic variation play a critical role in tumor development, progression and therapeutic resistance (23–25). These include epigenetic alterations (e.g., DNA methylation, histone modifications) as well as transcriptomic variation – both of which operate at much faster rates than does the acquisition of genetic mutations, thus serving as substrates for evolution even in the absence of any genetic events (26, 27). Variation at the level of the genome, transcriptome, or epigenome can also contribute to tumor plasticity, i.e., the degree to which a tumor population can flexibly and reversibly transition cell states to respond to stress (28, 29) (**Figure 1C**). The presence and integration of both heterogeneity and plasticity within a tumor results in many possible evolutionary avenues for tumor growth and survival. Indeed, there is growing evidence of ‘dynamic phenotypic heterogeneity’, where cancer cells can be phenotypically ‘re-trained’ by chemotherapy, resulting in the induction of drug-tolerant states (25, 30–34).

It is increasingly evident that reducing evolution to an allele-centric framework incompletely captures the context in which evolution occurs (35). From the early stages of transformation to metastasis, cancer cells are exposed to an array of niche microenvironmental and therapeutic pressures - which in total impart selective forces that shape their genetic and phenotypic profiles. This results in billions of cancer cells that are locally optimized to have their own distinct cell states conducive to their survival. Further, cells can fluctuate among different metastable cell states, broadening the population’s effective phenotypic landscape, thereby increasing adaptive capacity. With this breathtaking diversity comes clear implications for how to improve upon current therapies, which typically manage to target only a proportion of all cell states within a tumor, resulting in the outgrowth of clones that circumvent therapy by retaining or adopting non-targeted cell states.

### Targeted Approaches Cannot Outpace Evolutionary Potential

Lack of tumor response or relapse has been noted in response to single agents (targeted therapy, immunotherapy) as well as combination chemotherapy, resulting in the ongoing search for second, third, and fourth-line agents in many cancers, despite their relative ineffectiveness (33–44). In contrast to the ‘scorched earth’ approach of chemotherapy, targeted therapies aim to spare normal cells by targeting specific cancer cell dependencies, driven by a single molecule or reliance on a certain cellular pathway (42). Similarly, ‘precision medicine’ seeks to rationally target individual branches, with their respective dependencies, within the evolutionary tree of a patient’s tumor. These approaches all rely on genetic characterization of tumors to identify therapeutic targets or biomarkers that predict tumor response to existing targeted therapy options (43, 44). Such agents include BRAF inhibitors, which selectively eliminate or inhibit the growth of cells that harbor *BRAF* mutations and imatinib, which specifically inhibits the aberrant tyrosine kinase produced by the *BCR-ABL* gene fusion in Philadelphia chromosome-positive chronic myelogenous leukemia (CML) (45).

Despite their promise, targeted approaches have their limitations. First, a single biopsy produces a restricted representation of the various niches occupied by a tumor and is unlikely to resolve the complete genomic landscape, with a recent study showing >100 million coding region mutations existing within a single tumor (33, 46–48). Given this, even if the predominant subclones harboring the detected molecular phenotype are targeted effectively, other subclones that are below the limit of detection and harbor different cellular dependencies may still survive and expand; indeed, this has been demonstrated by the suboptimal outcomes of patients treated with agents targeting sub-clonal driver mutations or copy number gains (18, 19). Second, sensitive subclones may also acquire therapeutic tolerance or resistance, subverting the effect of therapy and contributing to relapse and re-emergence of heterogeneity (**Figure 1D**). CML illustrates both of these points, as imatinib resistance has been shown to be due to pre-existing or acquired resistance (8, 9).

Further, focusing solely on genomic alterations neglects the contributions of non-genetic mechanisms of tumor resistance, which can be induced by therapy (29, 49). Recent studies demonstrate that in several cancer types, therapeutic intervention initiates cellular reprogramming that induces a drug-tolerant phenotype in the absence of a pre-existing resistant clone (13); in this case, continued targeted therapy may accelerate tumor progression. For instance, continued treatment with a BRAF inhibitor can cause metastasis of *RAS/BRAF*-mutant melanoma (48, 50). Further, treatment with EGFR and BRAF inhibitors in colorectal cancers can increase overall mutability and the likelihood of resistance, demonstrating that targeted therapy can transiently enhance evolutionary potential by accelerating genetic diversity (51).

The dominant framework for cancer resistance studies for the last 15 years has consisted of high-throughput sequencing analyses of pre- and/or post-treatment tumor biopsies. While highly informative, this approach is limited in its capacity to provide comprehensive understandings of the longitudinal evolutionary process in a tumor, specifically due to resolution limitations that preclude capture and tracking of rare clones over time. As a result, only sporadic snapshots of a cancer cell’s journey are captured following therapeutic exposure. Moreover, the design and development of targeted agents to date have largely been informed by genomic alteration measurements following therapy. However, the vast heterogeneity inherent to tumors and their unique evolutionary trajectories as they adapt to an assortment of microenvironments and respond to various stimuli are such that many evolutionary outcomes are possible for a given tumor. Indeed, recent single-cell studies have shown that multiple cell states are often present within a tumor and that different cell states can have different sensitivities to therapy (52). Therefore, therapeutic strategies to date may eliminate the majority of a tumor population, but certain subpopulations can survive and drive relapse (**Figure 1D**). Regardless of mechanism of action, it is unlikely that any single or combination of therapeutic agents can adequately address the large range of present and potential phenotypes (i.e., cellular states and dependencies) that can emerge across clones within a tumor. Thus, the rational next step will be to also reduce the total number of potential cell states and associated dependencies within a tumor.

## Functionalized Lineage Tracing Can Inform the Design and Monitoring of Homogenization Therapy

Intratumor heterogeneity has been consistently detected through numerous high-throughput genome/exome sequencing studies, thereby presenting a gene-centric view of evolution. However, due to the substantial number of cells within tumors (10^7^ to 10^12^) and sequencing error rate of traditional NGS-based methods, resolution is restricted to an allele frequency of approximately 0.1% (53), limiting our ability to resolve evolutionary dynamics of rare clones. By contrast, single-cell analyses enable characterization of intratumor heterogeneity at greater resolution. The challenge, now, is the linking of this high-resolution information to cell fate and clonal origin such that we gain a more complete understanding of therapeutic response and chemoresistance.

DNA barcoding approaches have been developed to allow the tracking of clones over time and the elucidation of clonal dynamics (54–56). Bhang et al. and Hata et al. were the first to use such an approach in cancer models, providing examples of rare pre-existing resistance as well as *de novo* acquisition of resistance driving therapeutic relapse (54, 55). However, these early approaches consisted of unidimensional measurements of barcode frequency, and could not enable further clonally-linked measurements for characterizing tumor heterogeneity and resistance.

**Figure 2:**
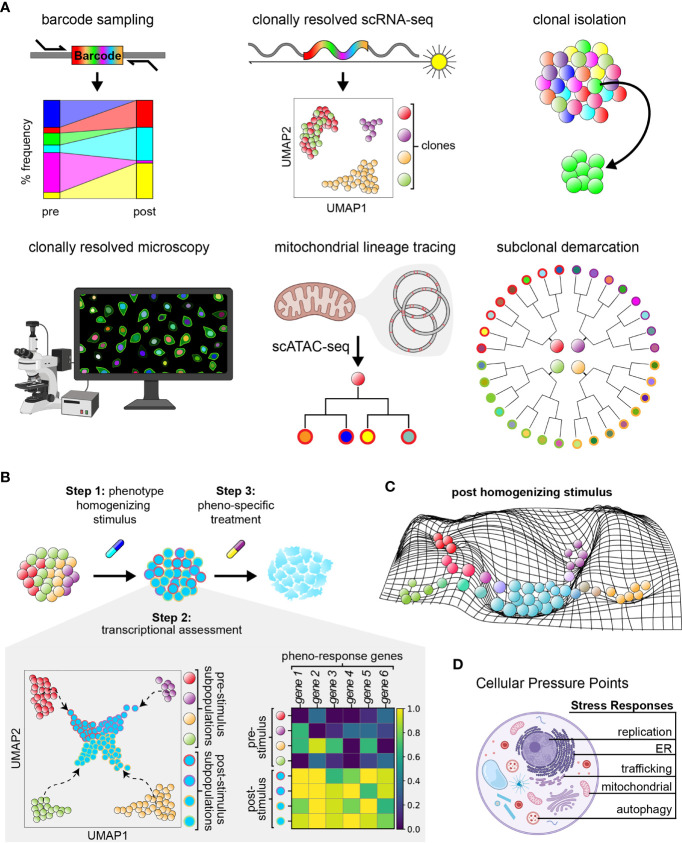
Functionalized lineage tracing can enable phenotypic homogenization. **(A)** Current features of multi-functionalized lineage tracing approaches. **(B)** Three–step model of phenotypic homogenization. Step 2 includes example uniform manifold approximations and projections (UMAP) of subpopulations within a cancer cell population prior to and after subjection to a phenotype homogenizing stimulus, as well as the corresponding matrix plot of differential gene expression data. Colors within the UMAP correspond to different cell states. Homogenized cells are outlined by colors representing their cell state of origin prior to treatment. Colors within the matrix plot represent the relative fold expression of each gene; yellow represents upregulation and dark blue represents downregulation of genes. Homogenized populations exhibit consistent upregulation of the same genes. **(C)** Example manifold depicting the phenotypic landscape of a cancer cell population shortly after treatment with a phenotype homogenizing stimulus. Cells of different cell state origins are pressured to adopt a new, more common phenotype. **(D)** Illustration of cellular stress responses of interest to target for achieving phenotypic homogenization.

As a result, functionalized lineage tracing approaches employing DNA barcodes have been more recently developed, allowing the linkage of clonal identity with transcriptomic features (e.g., CellTag, LARRY, Watermelon) (57–59), as well as the additional capacity to isolate and functionally characterize clones of interest for further multi-omic (Rewind) (60) and live cell analysis (e.g., ClonMapper, CloneSifter) (60, 61). Further, *in situ* lineage tracing methods have been developed, enabling integration of cellular profiling, spatial contexts, and clonal information (Rewind, intMEMOIR, Zombie) (60, 62). More recently, dynamic lineage-tracing systems, which enable sub-clonal demarcation over time, have been introduced and when paired with single-cell gene expression readouts have the potential to more deeply resolve clonal evolution (56, 63–68). These approaches have collectively permitted elucidation of the dynamic responses of heterogeneous populations to stimuli at clonal resolution across longitudinal phenotypic read-outs. Beyond engineered systems, lineage tracing in primary human samples has been possible by using mitochondrial mutations as native barcodes to enable multi-omic readout has also led to insights into clonal dynamics of therapeutic resistance in patients (**Figure 2A**) (69–71). Detailed and comprehensive reviews of existing lineage tracing systems with further information have been published (72) (see example approaches and their features, **Table 1**). Future studies using these tools will allow for exploration of the tumor-immune interface, spatial heterogeneity, *in vivo* clonal dynamics, and drug resistance and metastasis studies in primary cancer cells.

**Table 1:** Examples of Lineage Tracing Approaches and Their Features.

Year	Lineage Tracing Approach	Barcoding System	DNA barcode type	Clonal Read-out(s)	Notable Features	Citation
2015	ClonTracer	Lentiviral integration of 30-nucleotide S/W patterned DNA barcodes	Static	Targeted barcode sequencing		Bhang et al., Nature Medicine 2015 (54)
2019	Mitochondrial lineage tracing	Tracking of somatic mitochondrial DNA mutations as native genetic barcodes	Native	Single-cell RNA-sequencing, single-cell ATAC-sequencing	No cellular engineering necessary - mutations serve as native clonal inference markers	Ludwig et al., Cell 2019 (69)
2019	CellTag Indexing	Lentiviral integration of 8-nucleotide DNA barcodes; expressed within poly-adenylated transcripts	Static	Targeted barcode sequencing, single-cell RNA-sequencing		Guo et al., Genome Biology 2019 (57)
2020	LARRY	Lentiviral integration of 28-nucleotide DNA barcodes; expressed within poly-adenylated transcripts	Static	Targeted barcode sequencing, single-cell RNA-sequencing		Weinreb, Rodriguez-Fraticelli et al., Science 2020 (73)
2020	Zombie	Lentiviral integration of array of 20-nucleotide DNA barcodes. Barcodes are transcribed by phage RNA polymerases after fixation.	Evolving	RNA fluorescence in situ hybridization	Sub-clonal demarcation, spatial/morphological profiling	Askary et al., Nature Biotechnology 2020 (62)
2020	CloneSifter	Lentiviral integration of CRISPR sgRNA 20-nucleotide DNA barcodes; expressed within poly-adenylated transcripts (using CROPseq base vector)	Static	Targeted barcode sequencing, single-cell RNA sequencing	Live-cell clonal isolation	Feldman et al., BMC Biology 2020 (74)
2021	Target Site	Lentiviral or transposon-mediated integration of a static 14-nucleotide DNA barcode and 3 evolving Cas9-cut sites for recording; expressed within poly-adenylated transcripts	Evolving	Targeted barcode sequencing, single-cell RNA sequencing	Sub-clonal demarcation	Quinn et al., Science 2021 (63)
2021	IntMEMOIR	Integrase-mediated integration of array of 10 'memory elements' which can be irreversibly edited to generate heritable expressed DNA barcodes.	Evolving	RNA fluorescence in situ hybridization	In situ barcode detection, spatial/morphological profiling	Chow et al., Science 2021 (65)
2021	ClonMapper	Lentiviral integration of CRISPR sgRNA 20-nucleotide DNA barcodes expressed within poly-adenylated transcripts (using CROPseq base vector)	Static	Targeted barcode sequencing, single-cell RNA sequencing	Live-cell clonal isolation	Gutierrez et al., Nature Cancer 2021 (61)
2021	Rewind	Lentiviral integration of 100-nucleotide W/S/N patterned DNA barcodes expressed within poly-adenylated transcripts	Static	Single-cell RNA-sequencing, RNA fluorescence in situ hybridization	Fixed-cell clonal isolation, spatial/morphological profiling	Emert et al., Nature Biotechnology 2021 (60)
2021	Watermelon	Lentiviral integration of 30-nucleotide S/W patterned DNA barcodes expressed within poly-adenylated transcripts	Static	Single-cell RNA-sequencing	Enables tracking of proliferation	Oren et al., Nature 2021 (59)
2022	TraCe-Seq	Lentiviral integration of 30-nucleotide DNA barcodes expressed within poly-adenylated transcripts	Static	Targeted barcode sequencing, single-cell RNA sequencing		Chang et al., Nature Biotechnology 2022 (75)

## Phenotypic Homogenization: An Approach to Mitigating Intratumor Heterogeneity and Boosting Therapeutic Potential

While the concept of homogenization has been introduced in the literature (76), homogenization strategies are still in their infancy and require convincing experimental support. Several possible approaches have recently been proposed to reduce intratumoral heterogeneity and cancer cell plasticity. These include the targeting of shared pathways in settings where parallel mutations lead to pathway convergence (e.g., the PI3K/mTOR pathway in renal cancer, impacted by *PTEN*, *PIK3CA*, *TSC1* and *mTOR* mutations) (77), blocking cellular plasticity by preventing cell state transitions (e.g., inhibition of mediators of these processes: TGF-β and PI3K) (78), targeting the primary driver in a tumor while simultaneously blocking the anticipated adaptive response (e.g., PI3K inhibition in breast cancer can activate MAPK, thus motivating the combination of MEK and PI3K inhibitors) (79–81), or priming cancer cells with epigenetic drugs to sensitize them to subsequent treatment (e.g., with DNA methylation and HDAC inhibitors) (76, 82, 83). However, these approaches are challenged by the complexity of cellular signaling pathways, limitations in sequencing technologies, and difficulties in identifying the driver gene(s) amidst numerous passenger mutations within any given tumor (84, 85).

As an alternative, phenotypic homogenization, which involves creating an environment that serves to drive all tumor cells to exhibit a common targetable phenotype, is an attractive strategy (76). If achieved, it could then provide the backdrop against which subsequent administration of a drug targeting the shared phenotype of these cells could effectively eliminate the tumor population. Through this approach, it would be feasible for cells possessing disparate genetic backgrounds or residing in different transcriptomic and/or epigenetic niches to be confronted with a uniform potent stressor. Cells that fail to adequately sense and respond to the stressor would suffer a considerable negative fitness impact, while those that respond become reliant on a limited set of stress response pathways.

### Strategies for Tumor Homogenization

It is currently unknown what agents, targetable states, and to what extent homogenization is feasible. Conceptually, the implementation of tumor homogenization could be systematically pursued through a three step process (**Figures 2B, C** **)**. First is homogenization: as described by Tong et al., a selective pressure (i.e., therapeutic agent or combination of agents) can be introduced, coercing all tumor cells to exhibit a common phenotype that is vulnerable to a second agent which would eliminate the entire tumor population (also termed ‘collateral sensitivity’) (76, 86). Indeed, a recent study demonstrated that development of resistance to dasatinib treatment induces collateral sensitivity to non-classical BCR-ABL inhibitors, cabozantinib and vandetanib, in a murine model of acute lymphoid leukemia (87–89). Second is characterization of the homogenized state: extent of phenotypic homogenization within a tumor can be assessed through methods such as single-cell RNA-sequencing. Third is targeting of the homogenized state: therapeutic agents should be identified which can either eliminate the homogenized population through targeting of the shared phenotypic state, or funnel the homogenized cancer populations further into a defined targetable or sensitized state for elimination. Testing and selection of existing therapeutic agents at approved physiological doses would enable ease of clinical implementation.

The feasibility of homogenization strategies was first successfully tested in yeast (90), demonstrating that evolutionary dynamics can be manipulated for homogenization therapy. In support of the efficacy of homogenization therapy, a case study of a patient with ALK-rearranged non-small-cell lung cancer (NSCLC) has been described, where the authors postulated that cells exhibited unique, temporally restricted collateral sensitivities during adaptation to ALK inhibition (49, 91). Additionally, prior patient studies similarly have shown that convergent evolution in response to therapy is possible (16, 92, 93). The concept that cancer cell populations can be therapeutically modulated to transform cellular plasticity into therapeutic opportunities has been recently described in practice (49, 93, 94). For example, Frede et al. found that myeloma cells can modulate lineage restriction, adapt their enhancer usage, and employ cell-intrinsic diversity for survival and treatment escape, resulting in the co-existence of numerous distinct transcriptional states (95). Further, they demonstrated that standard therapy promotes transcriptional reprogramming while simultaneously reducing developmental potential, resulting in actionable immunotherapy targets (e.g., CXCR4) that could be exploited to overcome resistance (95). In another study, Lin et al. demonstrated that drug-induced antagonistic pleiotropy, the concept that genes can induce opposite effects on fitness in response to different drugs, can be leveraged to identify evolutionary traps which selectively target therapeutic resistance (96).

While cancer cells often rely on multiple stress response pathways to evade apoptosis and survive harsh tumor environments (e.g., the integrated stress response, cytosolic heat shock response, and unfolded protein response mediated by organelles such as the endoplasmic reticulum and mitochondria), induction of these cellular processes have also been noted to contribute to drug sensitivity of cancer cells. Activation of the integrated stress response in HER2+ breast cancer predicts a better response to trastuzumab therapy (97). In cancers with high protein turnover (e.g., multiple myeloma), agents that induce the unfolded protein response increase sensitivity to treatment with proteasome inhibitors through likely synergistic mechanisms (98). Further, cellular stress responses orchestrate common, potent responses across cells (i.e., *via* sweeping changes in cell state). For instance, ER stress induces the unfolded protein response, transducing multi-axis signaling and causing transcriptional reprogramming *via* IRE1α and ATF6, major translation modulation through phosphorylation of EIF2α, and pro-survival/-apoptotic signals dependent on resolution of ER stress (99, 100). In agreement, a recent lineage tracing study using TraCe-seq identified that efficacy of EGFR-inhibitor response is, in part, dependent on induction of ER stress (75). Similarly, replication stress has recently been described to activate immune-stimulating pathways, resulting in increased immune response to immunotherapies like PD-1/PDL-1 inhibitors across numerous cancer types, and serving as a reliable biomarker/predictor of clinical response to immune checkpoint blockade in patients (101, 102). For these reasons, induction of cellular stress responses may have great potential in actualizing phenotypic homogenization efforts (**Figure 2D**).

Homogenization strategies further require the ability to characterize cancer systems as they respond to sub-cytotoxic stress to uncover the nature of their responses, including the extent of their phenotypic uniformity and the duration of homogenization upon application and removal of stimulus. With the ongoing rapid development of multi-modal single-cell technologies, these characterizations will be greatly augmented. ClonMapper, Watermelon, and other dynamic expressed barcode systems enable one to distinguish how diverse clones differentially respond to therapy at single-cell resolution.

## Future Perspectives

The concept of tumor homogenization involves the induction of a ubiquitously adopted, targetable cell state across an initially heterogeneous cell population. Development of this approach is newly empowered by recent advances in lineage tracing techniques, which couple cell fate with multi-omic single-cell measurements and clonal isolation, enabling the identification and longitudinal monitoring of homogenized cancer cell states in detail. As newer multi-omic technologies with spatial resolution mature and innovative methods that approximate the complexity observed in primary tumors continue to be generated, we will be even better equipped to develop homogenization strategies. Homogenization therapy holds great promise as a generalizable strategy to anticipate and forestall evolutionary trajectories that lead to therapeutic resistance. Such a strategy enables a proactive rather than reactive approach to cancer therapy.

## Author Contributions

CG, CKV, and AMA wrote the manuscript. AMA and CJW revised the manuscript. AMA and CJW jointly oversaw this work. All authors read and approved the final manuscript.

## Funding

CG is supported by the Dana-Farber Cancer Institute Fellowship, the American Society of Hematology Minority Medical Student Award Program and the NIH Ruth L. Kirschstein NRSA Individual Predoctoral Fellowship F31 Award (1F31CA239443-01). CKV is supported by the NIH Ruth L. Kirschstein NRSA Individual Predoctoral Fellowship F31 Award (5F31CA243349-03) CJW is the Lavine Family Chair for Preventative Cancer Therapies at DFCI. AMA is supported by the Broad Institute IGNITE award.

## Conflict of Interest

CJW receives research funding from Pharmacyclics, and is an equity holder of BioNTech, Inc.

The remaining authors declare that the research was conducted in the absence of any commercial or financial relationships that could be construed as a potential conflict of interest.

## Publisher’s Note

All claims expressed in this article are solely those of the authors and do not necessarily represent those of their affiliated organizations, or those of the publisher, the editors and the reviewers. Any product that may be evaluated in this article, or claim that may be made by its manufacturer, is not guaranteed or endorsed by the publisher.

## References

[1] Greaves M Maley CC. Clonal Evolution in Cancer. Nature (2012) 481(7381):306–13. doi: 10.1038/nature10762 PMC3367003 22258609

[2] Marusyk A Janiszewska M Polyak K. Intratumor Heterogeneity: The Rosetta Stone of Therapy Resistance. Cancer Cell (2020) 37(4):471–84. doi: 10.1016/j.ccell.2020.03.007 PMC7181408 32289271

[3] Suzuki H Nakane S. Differential Induction of Chromosomal Aberrations by Topoisomerase Inhibitors in Cultured Chinese Hamster Cells. Biol Pharm Bull (1994) 17(2):222–6. doi: 10.1248/bpb.17.222 8205120

[4] Boot A Huang MN Ng AWT Ho S-C Lim JQ Kawakami Y. In-Depth Characterization of the Cisplatin Mutational Signature in Human Cell Lines and in Esophageal and Liver Tumors. Genome Res (2018) 28(5):654–65. doi: 10.1101/gr.230219.117 PMC5932606 29632087

[5] Szikriszt B Póti Á Pipek O Krzystanek M Kanu N Molnár J. A Comprehensive Survey of the Mutagenic Impact of Common Cancer Cytotoxics. Genome Biol (2016) 17(1):99. doi: 10.1186/s13059-016-0963-7 27161042 PMC4862131

[6] Ouzon-Shubeita H Vilas CK Lee S. Structural Insights Into the Promutagenic Bypass of the Major Cisplatin-Induced DNA Lesion. Biochem J (2020) 477(5):937–51. doi: 10.1042/BCJ20190906 PMC7534533 32039434

[7] Howlader N Noone AM Krapcho M Miller D Brest A Yu M. SEER Cancer Statistics Review, 1975-2017. Bethesda, MD: National Cancer Institute (2020).

[8] Valent P. Imatinib-Resistant Chronic Myeloid Leukemia (CML): Current Concepts on Pathogenesis and New Emerging Pharmacologic Approaches. Biol Targets Ther (2007) 1(4):433–48. PMC2721289 19707313

[9] O’Hare T Zabriskie MS Eiring AM Deininger MW. Pushing the Limits of Targeted Therapy in Chronic Myeloid Leukaemia. Nat Rev Cancer (2012) 12(8):513–26. doi: 10.1038/nrc3317 22825216

[10] Lee JH Shklovskaya E Lim SY Carlino MS Menzies AM Stewart A. Transcriptional Downregulation of MHC Class I and Melanoma De- Differentiation in Resistance to PD-1 Inhibition. Nat Commun (2020) 11(1):1897. doi: 10.1038/s41467-020-15726-7 32312968 PMC7171183

[11] Koyama S Akbay EA Li YY Herter-Sprie GS Buczkowski KA Richards WG. Adaptive Resistance to Therapeutic PD-1 Blockade Is Associated With Upregulation of Alternative Immune Checkpoints. Nat Commun (2016) 7:10501. doi: 10.1038/ncomms10501 26883990 PMC4757784

[12] Nam AS Chaligne R Landau DA. Integrating Genetic and Non-Genetic Determinants of Cancer Evolution by Single-Cell Multi-Omics. Nat Rev Genet (2021) 22(1):3–18. doi: 10.1038/s41576-020-0265-5 32807900 PMC8450921

[13] Raghavan S Winter PS Navia AW Williams HL DenAdel A Lowder KE. Microenvironment Drives Cell State, Plasticity, and Drug Response in Pancreatic Cancer. Cell (2021) 184(25):6119–37.e26. doi: 10.1016/j.cell.2021.11.017 PMC8822455 34890551

[14] Andor N Graham TA Jansen M Xia LC Aktipis CA Petritsch C. Pan-Cancer Analysis of the Extent and Consequences of Intratumor Heterogeneity. Nat Med (2016) 1):105–13. doi: 10.1038/nm.3984 PMC4830693 26618723

[15] Turajlic S Xu H Litchfield K Rowan A Chambers T Lopez JI. Tracking Cancer Evolution Reveals Constrained Routes to Metastases: TRACERx Renal. Cell (2018) 173(3):581–94:e12. doi: 10.1016/j.cell.2018.03.057 PMC5938365 29656895

[16] McGranahan N Swanton C. Clonal Heterogeneity and Tumor Evolution: Past, Present, and the Future. Cell (2017) 168(4):613–28. doi: 10.1016/j.cell.2017.01.018 28187284

[17] Mroz EA Rocco JW. MATH, a Novel Measure of Intratumor Genetic Heterogeneity, is High in Poor-Outcome Classes of Head and Neck Squamous Cell Carcinoma. Oral Oncol (2013) 49(3):211–5. doi: 10.1016/j.oraloncology.2012.09.007 PMC3570658 23079694

[18] Zhang J Fujimoto J Zhang J Wedge DC Song X Zhang J. Intratumor Heterogeneity in Localized Lung Adenocarcinomas Delineated by Multiregion Sequencing. Science (2014) 346(6206):256–9. doi: 10.1126/science.1256930 PMC4354858 25301631

[19] Schwarz RF Ng CKY Cooke SL Newman S Temple J Piskorz AM. Spatial and Temporal Heterogeneity in High-Grade Serous Ovarian Cancer: A Phylogenetic Analysis. PloS Med (2015) 12(2):e1001789. doi: 10.1371/journal.pmed.1001789 25710373 PMC4339382

[20] Davis A Gao R Navin N. Tumor Evolution: Linear, Branching, Neutral or Punctuated? Biochim Biophys Acta 1867(2):151–61. doi: 10.1016/j.bbcan.2017.01.003 PMC5558210 28110020

[21] Sottoriva A Kang H Ma Z Graham TA Salomon MP Zhao J. A Big Bang Model of Human Colorectal Tumor Growth. Nat Genet (2015) 47(3):209–16. doi: 10.1038/ng.3214 PMC4575589 25665006

[22] Gutierrez C Wu CJ. Clonal Dynamics in Chronic Lymphocytic Leukemia. Blood Adv (2019) 3(22):3759–69. doi: 10.1182/bloodadvances.2019000367 PMC6880909 31770443

[23] Flavahan WA Gaskell E Bernstein BE. Epigenetic Plasticity and the Hallmarks of Cancer. Science (2017) 357(6348):eaal2380. doi: 10.1126/science.aal2380 28729483 PMC5940341

[24] Bell CC Gilan O. Principles and Mechanisms of Non-Genetic Resistance in Cancer. Br J Cancer (2020) 122(4):465–72. doi: 10.1038/s41416-019-0648-6 PMC7028722 31831859

[25] Pisco AO Brock A Zhou J Moor A Mojtahedi M Jackson D. Non-Darwinian Dynamics in Therapy-Induced Cancer Drug Resistance. Nat Commun (2013) 4(1):2467. doi: 10.1038/ncomms3467 24045430 PMC4657953

[26] Brown R Curry E Magnani L Wilhelm-Benartzi CS Borley J. Poised Epigenetic States and Acquired Drug Resistance in Cancer. Nat Rev Cancer (2014) 14(11):747–53. doi: 10.1038/nrc3819 25253389

[27] Jones PA Baylin SB. The Epigenomics of Cancer. Cell (2007) 128(4):683–92. doi: 10.1016/j.cell.2007.01.029 PMC3894624 17320506

[28] Gunnarsson EB De S Leder K Foo J. Understanding the Role of Phenotypic Switching in Cancer Drug Resistance. J Theor Biol (2020) 490:110162. doi: 10.1016/j.jtbi.2020.110162 31953135 PMC7785289

[29] Marine J-C Dawson S-J Dawson MA. Non-Genetic Mechanisms of Therapeutic Resistance in Cancer. Nat Rev Cancer (2020) 20(12):743–56. doi: 10.1038/s41568-020-00302-4 33033407

[30] Goldman A Majumder B Dhawan A Ravi S Goldman D Kohandel M. Temporally Sequenced Anticancer Drugs Overcome Adaptive Resistance by Targeting a Vulnerable Chemotherapy-Induced Phenotypic Transition. Nat Commun (2015) 6(1):6139. doi: 10.1038/ncomms7139 25669750 PMC4339891

[31] Ravindran Menon D Das S Krepler C Vultur A Rinner B Schauer S. A Stress-Induced Early Innate Response Causes Multidrug Tolerance in Melanoma. Oncogene (2015) 34(34):4448–59. doi: 10.1038/onc.2014.372 PMC4442085 25417704

[32] Dallas NA Xia L Fan F Gray MJ Gaur P van Buren G. Chemoresistant Colorectal Cancer Cells, the Cancer Stem Cell Phenotype, and Increased Sensitivity to Insulin-Like Growth Factor-I Receptor Inhibition. Cancer Res (2009) 69(5):1951–7. doi: 10.1158/0008-5472.CAN-08-2023 PMC3198868 19244128

[33] Pirozzi G Tirino V Camerlingo R Franco R La Rocca A Liguori E. Epithelial to Mesenchymal Transition by Tgfβ-1 Induction Increases Stemness Characteristics in Primary Non Small Cell Lung Cancer Cell Line. PloS One (2011) 6(6):e21548. doi: 10.1371/journal.pone.0021548 21738704 PMC3128060

[34] Gupta PB Fillmore CM Jiang G Shapira SD Tao K Kuperwasser C. Stochastic State Transitions Give Rise to Phenotypic Equilibrium in Populations of Cancer Cells. Cell (2011) 146(4):633–44. doi: 10.1016/j.cell.2011.07.026 21854987

[35] Scott J Marusyk A. Somatic Clonal Evolution: A Selection-Centric Perspective. Biochim Biophys Acta Rev Cancer (2017) 1867(2):139–50. doi: 10.1016/j.bbcan.2017.01.006 28161395

[36] Bukowski K Kciuk M Kontek R. Mechanisms of Multidrug Resistance in Cancer Chemotherapy. Int J Mol Sci (2020) 21(9):3233. doi: 10.3390/ijms21093233 PMC7247559 32370233

[37] Alfarouk KO Stock C-M Taylor S Walsh M Muddathir AK Verduzco D. Resistance to Cancer Chemotherapy: Failure in Drug Response From ADME to P-Gp. Cancer Cell Int (2015) 15(1):71. doi: 10.1186/s12935-015-0221-1 26180516 PMC4502609

[38] Faltas BM Prandi D Tagawa ST Molina AM Nanus DM Sternberg C. Clonal Evolution of Chemotherapy-Resistant Urothelial Carcinoma. Nat Genet (2016) 48(12):1490–9. doi: 10.1038/ng.3692 PMC5549141 27749842

[39] Landau DA Tausch E Taylor-Weiner AN Stewart C Reiter JG Bahlo J. Mutations Driving CLL and Their Evolution in Progression and Relapse. Nature. (2015) 526(7574):525–30. doi: 10.1038/nature15395 PMC4815041 26466571

[40] Blakemore SJ Clifford R Parker H Antoniou P Stec-Dziedzic E Larrayoz M. Clinical Significance of TP53, BIRC3, ATM and MAPK-ERK Genes in Chronic Lymphocytic Leukaemia: Data From the Randomised UK LRF CLL4 Trial. Leukemia (2020) 34(7):1760–74. doi: 10.1038/s41375-020-0723-2 PMC7326706 32015491

[41] Pisco AO Huang S. Non-Genetic Cancer Cell Plasticity and Therapy-Induced Stemness in Tumour Relapse: ‘What Does Not Kill Me Strengthens Me. Br J Cancer (2015) 112(11):1725–32. doi: 10.1038/bjc.2015.146 PMC4647245 25965164

[42] Zhong L Li Y Xiong L Wang W Wu M Yuan T. Small Molecules in Targeted Cancer Therapy: Advances, Challenges, and Future Perspectives. Signal Transduct Target Ther (2021) 6(1):1–48. doi: 10.1038/s41392-021-00572-w 34054126 PMC8165101

[43] Sun C Fang Y Yin J Chen J Ju Z Zhang D. Rational Combination Therapy With PARP and MEK Inhibitors Capitalizes on Therapeutic Liabilities in RAS Mutant Cancers. Sci Transl Med (2017) 9(392):eaal5148. doi: 10.1126/scitranslmed.aal5148 28566428 PMC5919217

[44] Moss TJ Qi Y Xi L Peng B Kim T-B Ezzedine NE. Comprehensive Genomic Characterization of Upper Tract Urothelial Carcinoma. Eur Urol (2017) 4):641–9. doi: 10.1016/j.eururo.2017.05.048 28601352

[45] An X Tiwari AK Sun Y Ding P-R Ashby CR Chen Z-S. BCR-ABL Tyrosine Kinase Inhibitors in the Treatment of Philadelphia Chromosome Positive Chronic Myeloid Leukemia: A Review. Leuk Res (2010) 34(10):1255–68. doi: 10.1016/j.leukres.2010.04.016 20537386

[46] Ling S Hu Z Yang Z Yang F Li Y Lin P. Extremely High Genetic Diversity in a Single Tumor Points to Prevalence of non-Darwinian Cell Evolution. Proc Natl Acad Sci (2015) 112(47):E6496–505. doi: 10.1073/pnas.1519556112 PMC4664355 26561581

[47] Lohr JG Stojanov P Carter SL Cruz-Gordillo P Lawrence MS Auclair D. Widespread Genetic Heterogeneity in Multiple Myeloma: Implications for Targeted Therapy. Cancer Cell (2014) 25(1):91–101. doi: 10.1016/j.ccr.2013.12.015 24434212 PMC4241387

[48] Pearson A Smyth E Babina IS Herrera-Abreu MT Tarazona N Peckitt C. High-Level Clonal FGFR Amplification and Response to FGFR Inhibition in a Translational Clinical Trial. Cancer Discov (2016) 6(8):838–51. doi: 10.1158/2159-8290.CD-15-1246 PMC5338732 27179038

[49] Vander Velde R Yoon N Marusyk V Durmaz A Dhawan A Miroshnychenko D. Resistance to Targeted Therapies as a Multifactorial, Gradual Adaptation to Inhibitor Specific Selective Pressures. Nat Commun (2020) 11(1):2393. doi: 10.1038/s41467-020-16212-w 32409712 PMC7224215

[50] BRAF Inhibitors Induce Metastasis in RAS Mutant or Inhibitor-Resistant Melanoma Cells by Reactivating MEK and ERK Signaling [Internet]. [Cited 2021 Nov 11]. 10.1126/scisignal.2004815 24667377

[51] Russo M Crisafulli G Sogari A Reilly NM Arena S Lamba S. Adaptive Mutability of Colorectal Cancers in Response to Targeted Therapies. Science (2019) 366(6472):1473–80. doi: 10.1126/science.aav4474 31699882

[52] Neftel C Laffy J Filbin MG Hara T Shore ME Rahme GJ. An Integrative Model of Cellular States, Plasticity, and Genetics for Glioblastoma. Cell (2019) 178(4):835–49.e21. doi: 10.1016/j.cell.2019.06.024 PMC6703186 31327527

[53] Robasky K Lewis NE Church GM. The Role of Replicates for Error Mitigation in Next-Generation Sequencing. Nat Rev Genet (2014) 15(1):56–62. doi: 10.1038/nrg3655 24322726 PMC4103745

[54] Bhang HC Ruddy DA Krishnamurthy Radhakrishna V Caushi JX Zhao R Hims MM. Studying Clonal Dynamics in Response to Cancer Therapy Using High-Complexity Barcoding. Nat Med (2015) 21(5):440–8. doi: 10.1038/nm.3841 25849130

[55] Hata AN Niederst MJ Archibald HL Gomez-Caraballo M Siddiqui FM Mulvey HE. Tumor Cells can Follow Distinct Evolutionary Paths to Become Resistant to Epidermal Growth Factor Receptor Inhibition. Nat Med (2016) 22(3):262–9. doi: 10.1038/nm.4040 PMC4900892 26828195

[56] Pei W Feyerabend TB Rössler J Wang X Postrach D Busch K. Polylox Barcoding Reveals Haematopoietic Stem Cell Fates Realized *In Vivo* . Nature (2017) 548(7668):456–60. doi: 10.1038/nature23653 PMC5905670 28813413

[57] Guo C Kong W Kamimoto K Rivera-Gonzalez GC Yang X Kirita Y. CellTag Indexing: Genetic Barcode-Based Sample Multiplexing for Single-Cell Genomics. Genome Biol (2019) 20(1):90. doi: 10.1186/s13059-019-1699-y 31072405 PMC6509836

[58] Tang L. Integrating Lineage Tracing and Single-Cell Analysis. Nat Methods (2020) 4):359–9. doi: 10.1038/s41592-020-0802-3 32242155

[59] Oren Y Tsabar M Cuoco MS Amir-Zilberstein L Cabanos HF Hütter J-C. Cycling Cancer Persister Cells Arise From Lineages With Distinct Programs. Nature (2021) 596(7873):576–82. doi: 10.1038/s41586-021-03796-6 PMC9209846 34381210

[60] Emert BL Cote CJ Torre EA Dardani IP Jiang CL Jain N. Variability Within Rare Cell States Enables Multiple Paths Toward Drug Resistance. Nat Biotechnol (2021) 39(7):865–76. doi: 10.1038/s41587-021-00837-3 PMC8277666 33619394

[61] Gutierrez C Al’Khafaji AM Brenner E Johnson KE Gohil SH Lin Z. Multifunctional Barcoding With ClonMapper Enables High-Resolution Study of Clonal Dynamics During Tumor Evolution and Treatment. Nat Cancer (2021) 2(7):758–72. doi: 10.1038/s43018-021-00222-8 PMC8691751 34939038

[62] Askary A Sanchez-Guardado L Linton JM Chadly DM Budde MW Cai L. *In Situ* Readout of DNA Barcodes and Single Base Edits Facilitated by *In Vitro* Transcription. Nat Biotechnol (2020) 38(1):66–75. doi: 10.1038/s41587-019-0299-4 31740838 PMC6954335

[63] Quinn JJ Jones MG Okimoto RA Nanjo S Chan MM Yosef N. Single-Cell Lineages Reveal the Rates, Routes, and Drivers of Metastasis in Cancer Xenografts. Science 371(6532):eabc1944. doi: 10.1126/science.abc1944 PMC7983364 33479121

[64] Yang D Jones MG Naranjo S Rideout WM Min KHJ Ho R. Lineage Recording Reveals the Phylodynamics, Plasticity and Paths of Tumor Evolution [Internet]. 2021 Oct [Cited 2021 Nov 12]. (2021). doi: 10.1101/2021.10.12.464111

[65] Chow K-HK Budde MW Granados AA Cabrera M Yoon S Cho S. Imaging Cell Lineage With a Synthetic Digital Recording System. Science (2021) 372(6538):eabb3099. doi: 10.1126/science.abb3099 33833095

[66] Bowling S Sritharan D Osorio FG Nguyen M Cheung P Rodriguez-Fraticelli A. An Engineered CRISPR-Cas9 Mouse Line for Simultaneous Readout of Lineage Histories and Gene Expression Profiles in Single Cells. Cell (2020) 181(6):1410–22.e27. doi: 10.1016/j.cell.2020.04.048 PMC7529102 32413320

[67] Raj B Wagner DE McKenna A Pandey S Klein AM Shendure J. Simultaneous Single-Cell Profiling of Lineages and Cell Types in the Vertebrate Brain. Nat Biotechnol (2018) 36(5):442–50. doi: 10.1038/nbt.4103 PMC5938111 29608178

[68] Loveless TB Grotts JH Schechter MW Forouzmand E Carlson CK Agahi BS. Lineage Tracing and Analog Recording in Mammalian Cells by Single-Site DNA Writing. Nat Chem Biol (2021) 17(6):739–47. doi: 10.1038/s41589-021-00769-8 PMC8891441 33753928

[69] Ludwig LS Lareau CA Ulirsch JC Christian E Muus C Li LH. Lineage Tracing in Humans Enabled by Mitochondrial Mutations and Single-Cell Genomics. Cell (2019) 176(6):1325–39.e22. doi: 10.1016/j.cell.2019.01.022 PMC6408267 30827679

[70] Xu J Nuno K Litzenburger UM Qi Y Corces MR Majeti R. Single-Cell Lineage Tracing by Endogenous Mutations Enriched in Transposase Accessible Mitochondrial DNA. eLife (2019) 8:e45105. doi: 10.7554/eLife.45105 30958261 PMC6469926

[71] Miller TE Lareau CA Verga JA DePasquale EAK Liu V Ssozi D. Mitochondrial Variant Enrichment From High-Throughput Single-Cell RNA Sequencing Resolves Clonal Populations. Nat Biotechnol (2022). doi: 10.1038/s41587-022-01210-8 PMC9288977 35210612

[72] VanHorn S Morris SA. Next-Generation Lineage Tracing and Fate Mapping to Interrogate Development. Dev Cell (2021) 56(1):7–21. doi: 10.1016/j.devcel.2020.10.021 33217333

[73] Weinreb C Rodriguez-Fraticelli A Camargo FD Klein AM. Lineage Tracing on Transcriptional Landscapes Links State to Fate During Differentiation. Science (2020) 367(6479):eaaw3381. doi: 10.1126/science.aaw3381 31974159 PMC7608074

[74] Feldman D Tsai F Garrity AJ O’Rourke R Brenan L Ho P. CloneSifter: Enrichment of Rare Clones from Heterogeneous Cell Populations. BMC Biol (2020) 18(177). doi: 10.1186/s12915-020-00911-3 PMC7687773 33234154

[75] Chang MT Shanahan F Nguyen TTT Staben ST Gazzard L Yamazoe S. Identifying Transcriptional Programs Underlying Cancer Drug Response With TraCe-Seq. Nat Biotechnol (2022) 40(1):86–93. doi: 10.1038/s41587-021-01005-3 34531539

[76] Tong M Deng Z Zhang X He B Yang M Cheng W. New Insights From the Widening Homogeneity Perspective to Target Intratumor Heterogeneity. Cancer Commun (2018) 38:17. doi: 10.1186/s40880-018-0287-y PMC5993146 29764517

[77] Voss MH Hakimi AA Pham CG Brannon AR Chen Y-B Cunha LF. Tumor Genetic Analyses of Patients With Metastatic Renal Cell Carcinoma and Extended Benefit From mTOR Inhibitor Therapy. Clin Cancer Res Off J Am Assoc Cancer Res (2014) 20(7):1955–64. doi: 10.1158/1078-0432.CCR-13-2345 PMC4140619 24622468

[78] Bhola NE Balko JM Dugger TC Kuba MG Sánchez V Sanders M. TGF-β Inhibition Enhances Chemotherapy Action Against Triple-Negative Breast Cancer. J Clin Invest (2013) 123(3):1348–58. doi: 10.1172/JCI65416 PMC3582135 23391723

[79] Zardavas D Irrthum A Swanton C Piccart M. Clinical Management of Breast Cancer Heterogeneity. Nat Rev Clin Oncol (2015) 12(7):381–94. doi: 10.1038/nrclinonc.2015.73 25895611

[80] Shimizu T Tolcher AW Papadopoulos KP Beeram M Rasco DW Smith LS. The Clinical Effect of the Dual-Targeting Strategy Involving PI3K/AKT/mTOR and RAS/MEK/ERK Pathways in Patients With Advanced Cancer. Clin Cancer Res Off J Am Assoc Cancer Res (2012) 18(8):2316–25. doi: 10.1158/1078-0432.CCR-11-2381 22261800

[81] Mirzoeva OK Das D Heiser LM Bhattacharya S Siwak D Gendelman R. Basal Subtype and MAPK/ERK Kinase (MEK)-Phosphoinositide 3-Kinase Feedback Signaling Determine Susceptibility of Breast Cancer Cells to MEK Inhibition. Cancer Res (2009) 69(2):565–72. doi: 10.1158/0008-5472.CAN-08-3389 PMC2737189 19147570

[82] Baylin SB Jones PA. A Decade of Exploring the Cancer Epigenome — Biological and Translational Implications. Nat Rev Cancer (2011) 11(10):726–34. doi: 10.1038/nrc3130 PMC3307543 21941284

[83] Azad N Zahnow CA Rudin CM Baylin SB. The Future of Epigenetic Therapy in Solid Tumours–Lessons From the Past. Nat Rev Clin Oncol (2013) 10(5):256–66. doi: 10.1038/nrclinonc.2013.42 PMC3730253 23546521

[84] Klempner SJ Myers AP Cantley LC. What a Tangled Web We Weave: Emerging Resistance Mechanisms to Inhibition of the Phosphoinositide 3-Kinase Pathway. Cancer Discov (2013) 3(12):1345–54. doi: 10.1158/2159-8290.CD-13-0063 PMC3864542 24265156

[85] Duncan JS Whittle MC Nakamura K Abell AN Midland AA Zawistowski JS. Dynamic Reprogramming of the Kinome in Response to Targeted MEK Inhibition in Triple-Negative Breast Cancer. Cell (2012) 149(2):307–21. doi: 10.1016/j.cell.2012.02.053 PMC3328787 22500798

[86] Herencias C Rodríguez-Beltrán J León-Sampedro R Alonso-del Valle A Palkovičová J Cantón R. Collateral Sensitivity Associated With Antibiotic Resistance Plasmids. eLife (2021) 10:e65130. doi: 10.7554/eLife.65130 33470194 PMC7837676

[87] Jensen PB Holm B Sorensen M Christensen IJ Sehested M. *In Vitro* Cross-Resistance and Collateral Sensitivity in Seven Resistant Small-Cell Lung Cancer Cell Lines: Preclinical Identification of Suitable Drug Partners to Taxotere, Taxol, Topotecan and Gemcitabin. Br J Cancer (1997) 75(6):869–77. doi: 10.1038/bjc.1997.154 PMC2063407 9062409

[88] Hill BT. Potential of Continuous Tumour Cell Lines for Establishing Patterns of Cross-Resistance and Collateral Sensitivity *In Vitro* . Drugs Exp Clin Res (1986) 12(1–3):293–8. 3732052

[89] Zhao B Sedlak JC Srinivas R Creixell P Pritchard JR Tidor B. Exploiting Temporal Collateral Sensitivity in Tumor Clonal Evolution. Cell (2016) 165(1):234–46. doi: 10.1016/j.cell.2016.01.045 PMC5152932 26924578

[90] Chen G Mulla WA Kucharavy A Tsai H-J Rubinstein B Conkright J. Targeting the Adaptability of Heterogeneous Aneuploids. Cell (2015) 160(4):771–84. doi: 10.1016/j.cell.2015.01.026 PMC4328141 25679766

[91] Shaw AT Friboulet L Leshchiner I Gainor JF Bergqvist S Brooun A. Resensitization to Crizotinib by the Lorlatinib ALK Resistance Mutation L1198F. N Engl J Med (2016) 374(1):54–61. doi: 10.1056/NEJMoa1508887 26698910 PMC4773904

[92] Johnson BE Mazor T Hong C Barnes M Aihara K McLean CY. Mutational Analysis Reveals the Origin and Therapy-Driven Evolution of Recurrent Glioma. Science (2014) 343(6167):189–93. doi: 10.1126/science.1239947 PMC3998672 24336570

[93] Singleton KR Crawford L Tsui E Manchester HE Maertens O Liu X. Melanoma Therapeutic Strategies That Select Against Resistance by Exploiting MYC-Driven Evolutionary Convergence. Cell Rep (2017) 21(10):2796–812. doi: 10.1016/j.celrep.2017.11.022 PMC5728698 29212027

[94] Wang L Leite de Oliveira R Huijberts S Bosdriesz E Pencheva N Brunen D. An Acquired Vulnerability of Drug-Resistant Melanoma With Therapeutic Potential. Cell (2018) 173(6):1413–25.e14. doi: 10.1016/j.cell.2018.04.012 29754815

[95] Frede J Anand P Sotudeh N Pinto RA Nair MS Stuart H. Dynamic Transcriptional Reprogramming Leads to Immunotherapeutic Vulnerabilities in Myeloma. Nat Cell Biol (2021) 23(11):1199–211. doi: 10.1038/s41556-021-00766-y PMC8764878 34675390

[96] Lin KH Rutter JC Xie A Pardieu B Winn ET Bello RD. Using Antagonistic Pleiotropy to Design a Chemotherapy-Induced Evolutionary Trap to Target Drug Resistance in Cancer. Nat Genet (2020) 52(4):408–17. doi: 10.1038/s41588-020-0590-9 PMC7398704 32203462

[97] Darini C Ghaddar N Chabot C Assaker G Sabri S Wang S. An Integrated Stress Response *via* PKR Suppresses HER2+ Cancers and Improves Trastuzumab Therapy. Nat Commun (2019) 10(1):2139. doi: 10.1038/s41467-019-10138-8 31086176 PMC6513990

[98] Obeng EA Carlson LM Gutman DM Harrington WJ Lee KP Boise LH. Proteasome Inhibitors Induce a Terminal Unfolded Protein Response in Multiple Myeloma Cells. Blood (2006) 107(12):4907–16. doi: 10.1182/blood-2005-08-3531 PMC1895817 16507771

[99] Galluzzi L Yamazaki T Kroemer G. Linking Cellular Stress Responses to Systemic Homeostasis. Nat Rev Mol Cell Biol (2018) 19(11):731–45. doi: 10.1038/s41580-018-0068-0 30305710

[100] Reich S Nguyen CDL Has C Steltgens S Soni H Coman C. A Multi-Omics Analysis Reveals the Unfolded Protein Response Regulon and Stress-Induced Resistance to Folate-Based Antimetabolites. Nat Commun (2020) 11(1):2936. doi: 10.1038/s41467-020-16747-y 32522993 PMC7287054

[101] McGrail DJ Pilié PG Dai H Lam TNA Liang Y Voorwerk L. Replication Stress Response Defects are Associated With Response to Immune Checkpoint Blockade in Nonhypermutated Cancers. Sci Transl Med (2021) 13(617):eabe6201. doi: 10.1126/scitranslmed.abe6201 34705519 PMC8577990

[102] Zeman MK Cimprich KA. Causes and Consequences of Replication Stress. Nat Cell Biol (2014) 16(1):2–9. doi: 10.1038/ncb2897 24366029 PMC4354890

